# Comment on “Transmission Model of Hepatitis B Virus with Migration Effect”

**DOI:** 10.1155/2015/492513

**Published:** 2015-12-17

**Authors:** Anwar Zeb, Gul Zaman

**Affiliations:** ^1^Department of Mathematics, COMSATS Institute of Information Technology Abbottabad, Khyber Pakhtunkhwa 22060, Pakistan; ^2^Department of Mathematics, University of Malakand Chakdara, Dir(L), Khyber Pakhtunkhwa 18800, Pakistan

## Abstract

We show the erroneous assumptions and reasoning by introducing the migration effect of individuals in the transmission model of Hepatitis B virus. First, some false results related to the eigenvalues and reproductive number in the recent literature in mathematical biology will be presented. Then, it will be proved that the product of the matrices in the next generation method to obtain the reproductive number *R*_0_ is not correct and the local and global stability results based on the reproductive number *R*_0_ are considered false.

## 1. Model Formulation

In this section, we consider the model developed by Khan et al. [1] taking into account migration class in the population by extending the work of Pang et al. [2]. In their work [1] they have categorized the model into six compartments: susceptible *S*(*t*), exposed *E*(*t*), acute *A*(*t*), carrier *C*(*t*), vaccinated *V*(*t*), and migrated *M*(*t*) individuals. On the basis of their assumptions what are the parameters in the model that bring out the migrated class? They have considered the following assumptions:

(i) the transmission rate *μ*_1_ from migrated class to exposed class,
(ii) the transmission rate *μ*_2_ from migrated to acute class,
(iii) the death rate *δ* of the migrated class.

The model presented in [1] is given by

(1) dStdt=δπ1−ηCt−δSt−βAt+kCtSt+δ0Vt−pSt,dEtdt=βAt+kCtSt−δEt+δπηCt−γ1Et+μ1Mt,dAtdt=γ1Et−δAt−qγ2At−1−qγ1At+μ2Mt,dCtdt=qγ2At−δCt−γ3Ct,dVtdt=γ3Ct+1−qγ1At−δ0Vt−δVt+δ1−π+pSt,dMtdt=−μ1+μ2Mt−δMt.

They have claimed that the migrated class of individuals come from different parts of the world to the host country, and their interaction occurs in the form of sexual interactions, blood transportation, and transfusion. What are the parameters in the model that bring out the migrated class? The authors have used in the new class (migrated) only three parameters: the transmission rate *μ*_1_ from migrated class to exposed class, the transmission rate *μ*_2_ from migrated to acute class, and the death rate *δ* of the migrated class. There is a serious flaw that how the migrated individuals move to different classes and there is no region and parameters defined in the model in which the population of migrated individuals have been generated. All the terms on the right hand side are negative in the migrated class of system (1); however, for large time it tends to zero. Since population is always positive (nonnegative), the said class (migrated) is not biologically feasible. Keeping these flaws in view the extended model of Khan et al. [1] is considered false and the goal is unlikely to be met if the conclusions of the model are based on false mathematical assumptions and reasoning.

## 2. Falseness of the Basic Reproduction Number in [1]

To find the basic reproductive number *R*_0_ the authors have used the concept of next generation method introduced by Van Den Driessche and Watmough [3]. Several authors have used this method to find the basic reproductive number for different epidemic models; see, for example, [2, 4, 5].

For the basic reproductive number *R*_0_ the authors in [1] considered that *F* and *V* are given by

(2) F=0βS0βkS0000000,V=δ+γ10−δπη−γ1δ+qγ2+1−qγ100−qω2δ+γ3.

The authors have used elementary matrix operation to find that *V* ^−1^ is given by

(3) V−1=δ+qγ2+1−qγ1δ+γ3qγ2δπηδπηδ+qγ2+1−qγ1γ1δ+γ3δ+γ1δ+γ3δπηγ1qγ1γ2qγ2δ+γ1δ+γ1δ+qγ2+1−qγ1δ+γ1δ+qγ2+1−qγ1δ+γ3−δπηqγ1γ2.

The authors have defined *Q*_1_ = (*δ* + *qγ*_2_ + (1 − *q*)*γ*_1_) but they did not use this value in the matrix.

The blunder of this paper is that the matrix multiplication *FV* ^−1^ is given by 
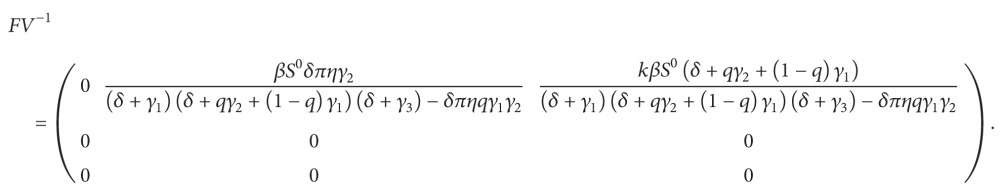
(4)The authors have obtained the above matrix by using matrix multiplication which is not correct. For simple matrix multiplication, see the following example.

**Example 1 .**

Let us consider the following two matrices:

(5) A=0a12a13000000,B=b11b12b13b21b22b23b31b32b33.

Then simple matrix multiplication of matrices *A* and *B* becomes

(6) AB=a12b21+a13b31a12b22+a12b32a12b23+a12b33000000.

Comparing the corresponding elements of *AB* and *FV* ^−1^, we conclude that the elements of *FV* ^−1^ are not correct and the correct one will be of the following form:

(7) $$\begin{array}{c} F^{*}{V^{*}}^{-1}=\left[\begin{array}{ccc} \frac{\beta S^{0}\gamma_{1}\left(\delta +\gamma_{3}\right)+k\beta S^{0}q\gamma_{1}\gamma_{2}}{\left|V\right|} & \frac{\beta S^{0}\left(\delta +\gamma_{1}\right)\left(\delta +\gamma_{3}\right)+k\beta S^{0}q\left(\delta +\gamma_{1}\right)\gamma_{2}}{\left|V\right|} & \frac{\text{Ł}}{\left|V\right|}\\ 0 & 0 & 0\\ 0 & 0 & 0 \end{array}\right], \end{array}$$

where

(8) Ł=−βS0δπηγ1+kβS0δ+γ1δ+qγ2+1−qγ1,V=δ+γ1δ+qγ2+1−qγ1δ+γ3−δπηqγ1γ2.

Now if we follow matrix *FV* ^−1^ of the authors, then all the eigenvalues become zero because all the terms on main diagonal of *FV* ^−1^ are zeros. This means that there exists no such reproductive number *R*_0_ which the authors have mentioned throughout the paper. However, if we follow *F* ^*∗*^ *V* ^*∗*^^−1^, then the reproductive number will be

(9) R0=βS0γ1δ+γ3+kβS0qγ1γ2δ+γ1δ+qγ2+1−qγ1δ+γ3−δπηqγ1γ2,

which is different from the reproductive number on page  4 in [1].

Also if we use the same method to find *F* and *V*, then the correct matrices become

(10) F=0βS0βkS0+δπη000000,V=δ+γ100−γ1δ+qγ2+1−qγ100−qω2δ+γ3.

Thus the reproductive number will be

(11) $$\begin{array}{c} R_{0}=\frac{\gamma_{1}\beta S^{0}\left(\delta +\gamma_{3}\right)+q\gamma_{1}\gamma_{2}\left(\beta kS^{0}+\delta \pi \eta \right)}{\left(\delta +\gamma_{1}\right)\left(\delta +\gamma_{3}\right)Q_{1}}. \end{array}$$

If we follow this reproductive number, then the local stability will be completely determined by this reproductive number and no further condition is required.

**Theorem 2 .**

The disease-free equilibrium of the model (3) in [1] is locally asymptotically stable if *R*_0_ < 1 and unstable if *R*_0_ > 1.

For the endemic equilibria of the proposed model the authors have obtained *T* ^*∗*^ = (*S* ^*∗*^, *E* ^*∗*^, *A* ^*∗*^, *C* ^*∗*^, *M* ^*∗*^) by considering that the right hand side of each equation of system (3) in [1] is equal to zero. In order to follow this technique for endemic equilibria equation (6) in system (3) presented in [1]:

(12) $$\begin{array}{c} \frac{dM\left(t\right)}{dt}=0⟹-\left(\;\mu_{1}+\mu_{2}+\delta \;\right)M^{*}\left(\;t\;\right)=0. \end{array}$$

All parameters are nonnegative so their sum should always be positive. Thus, −*M* ^*∗*^ = 0 which is biologically not feasible and the objective of the paper [1] is based on baseless assumption. Therefore, all the results related to local and global stability presented in [1] are based on biologically not feasible and baseless assumption.

As every theorem about the local and global stability is based on *R*_0_ and their *R*_0_ is not correct, so this leads to incorrect proofs.

Beside this, the authors have used in [1] the Jacobian matrices for the local stability in (9); the second element *δ*_0_ of first row is positive and last element of first row is zero while these should be −*δ*_0_ in both.

Similarly in equation (10) in [1] last element of first row should be −*δ*_0_ and last term of fourth row is not zero that should be

(13) $$\begin{array}{c} -q\gamma_{2}\left(\;\mu_{2}\left(\;\delta +\sigma_{1}\;\right)+\gamma_{1}\mu_{1}\;\right). \end{array}$$

Also for the Jacobian matrix mentioned by equation (10) in [1] the authors have represented that

(14) T1=−−Q1δ+σ1+γ1βS0−qγ1γ2βkS0+δπη,

while this should be

(15) T1=−δ+σ3−Q1δ+σ1+γ1βS0−qγ1γ2βkS0+δπη.

In the same way one can follow (14) and (15) in [1].

## 3. Conclusion

The above discussion and reasoning indicates that conclusions based on the model studied by Khan et al. [1] may not be valid and migration of individuals may be possible to the host area but they should be in different forms. It may be possible that the migrated individuals already have got this type of infection, which means coming to the infected class, or may be possible to come to susceptible class and they do not make any type of interaction occuring in the form of sexual interactions, blood transportation, and transfusion. On the basis of this reasoning, migrated class mentioned in [1] is totally wrong, if someone wants to introduce the migrated people in the host area that should be based on the abovementioned assumptions.

Furthermore, the authors have mentioned in [1] on page 2 thrice, “we introduced the migrated class" which shows that the English presentation also needs correction. On page 3 the authors have written, “*γ*_2_ is the rate at which the individuals move to the carrier class,” while it should have written “*γ*_2_ be the rate at which the acute individuals move to the carrier class.” On the same page the authors have written, “*γ*_2_ is the rate of transmission from migrated class”; instead they should have written “*μ*_2_ which is the rate of transmission from migrated class to acute class.”
